# Human teratozoospermia-related *AGTPBP1* R791H mutation is associated with sperm head and tail defects in a CRISPR-engineered murine model

**DOI:** 10.1007/s10815-025-03732-x

**Published:** 2025-10-29

**Authors:** Ya-Yun Wang, Yu-Hua Lin, Chih-Chun Ke, Tsung-Hsuan Lai, I-Shing Yu, Chin-Fong Au, Rajender Singh, Ying-Hung Lin

**Affiliations:** 1 Graduate Institute of Biomedical and Pharmaceutical Science, Fu Jen Catholic University, New Taipei City, Taiwan; 2 Division of Urology, Department of Surgery, Cardinal Tien Hospital, New Taipe City, Taiwan; 3 Department of Chemistry, Fu Jen Catholic University, New Taipei City, Taiwan; 4 Department of Urology, En Chu Kong Hospital, New Taipei City, Taiwan; 5 Department of Obstetrics and Gynecology, Cathay General Hospital, Taipei, Taiwan; 6 Laboratory Animal Center, College of Medicine, National Taiwan University, Taipei, Taiwan; 7 Program in Nutrition & Food Science, Fu Jen Catholic University, New Taipei City, Taiwan; 8 Division of Urology, Department of Surgery, Far Eastern Memorial Hospital, New Taipei City, Taiwan; 9 Division of Endocrinology, CSIR-Central Drug Research Institute, Lucknow, India; 10 Academy of Scientific and Innovative Research (AcSIR), Ghaziabad, India

**Keywords:** Teratozoospermia, Genetic mutation, *Agtpbp1*, Knock-in mice, Sperm defect

## Abstract

**Background:**

Infertility is a pervasive global health concern affecting millions of couples worldwide. Approximately 7% of the male population is infertile. Teratozoospermia, defined by > 96% abnormal sperm morphology, is a major cause of infertility often linked to genetic defects. In our previous study, we identified three AGTPBP1 mutations (p.Glu423Asp, p.Pro631Leu, and p.Arg811His) in teratozoospermia cases. AGTPBP1 is a key enzyme involved in regulating tubulin polyglutamylation and generating Δ-2 tubulin, a major structural component of the sperm tail and an essential structure for sperm head differentiation. However, functional proof of the impact of AGTPBP1 Arg811His on sperm head and tail impairment remained unestablished.

**Methods:**

Knock-in mice carrying the equivalent mutation, Arg791His (R791H) corresponding to the human mutation (R811H), in the *Agtpbp1* gene were generated and analyzed for sperm morphological abnormalities.

**Results:**

Sperm morphological evaluation revealed a significant increase in the proportion of morphologically abnormal sperm in the *Agtpbp1*^*R791H/R791H*^ mice. Detailed morphological analysis revealed a significantly higher incidence of sperm head abnormalities and abnormal attachment of the head to the midpiece in the *Agtpbp1*^*R791H/R791H*^ mice relative to wild-type controls. Further, sperm with head defects from *Agtpbp1*^*R791H/R791H*^ mice exhibited abnormal accumulation of polyglutamylated tubulin within the sperm head. The mutant mice showed exactly the same morphological defects as seen in human patients and those displayed by mice lacking the complete carboxypeptidase A domain of AGTPBP1 but at a relatively lesser frequency.

**Conclusions:**

We conclude that the R791H mutation in the *Agtpbp1* gene impairs sperm head and tail differentiation, resulting in sperm morphological defects.

## Background

Male infertility affects approximately 7% of the male population, and its incidence is rising tremendously [1, 2]. Male infertility can result from a wide range of factors, including hormonal imbalances, anatomical abnormalities, lifestyle-related issues, psychological conditions, sexual dysfunction, chromosomal anomalies, and single-gene mutations [3–5]. In terms of sperm phenotype, infertility may be characterized by azoospermia, oligozoospermia, teratozoospermia, asthenozoospermia, or a combination of these. Teratozoospermia, defined by the World Health Organization as the presence of fewer than 4% morphologically normal sperm, is a key contributor to male infertility or subfertility [6–9]. Multiple factors have been implicated in the etiology of teratozoospermia, including infections, varicocele, environmental exposures, aneuploidy, gene mutations, and other underlying conditions, with genetic factors being the top contributors [8, 10–14]. Over the past decade, genetic investigations of teratozoospermia have become one of the most productive areas in male infertility research, driven by advances in genome-wide screening technologies [8, 15, 16]

AGTPBP1, predominantly expressed in the testes and brain, encodes an armadillo-type fold and a carboxypeptidase A domain-containing protein [17]. The carboxypeptidase A domain of AGTPBP1 exhibits enzymatic activity that specifically catalyzes the removal of polyglutamate residues from the C-terminus of alpha-tubulin (α-tubulin) [18, 19]. Additionally, this domain plays a pivotal role in the generation of delta-2 tubulin (△−2 tubulin), a stable form of tubulin. Recently, mutations in the *AGTPBP1* gene causing childhood-onset neurodegeneration with cerebellar atrophy (CONDCA) were characterized [18]. These mutations result in a reduction in deglutamylase activity and decreased levels of delta-2 tubulin (△−2 tubulin) [20]. The *Agtpbp1* allele deletion is identified in the spontaneous recessive *pcd* mouse strain, which exhibits Purkinje cell degeneration and male infertility [21–23]. Studies show that abnormal tubulin polyglutamylation in *pcd* neurons results from disrupted vesicular axonal transport, leading to neurodegeneration [24, 25]. Further, *pcd* mice also show male infertility due to increased apoptosis in male germ cells [23, 26].

Recently, we used whole-exome sequencing (WES) to identify novel genes related to teratozoospermia [15]. Three *AGTPBP1* non-synonymous mutations (E423D, P631L, and R811H) in infertile cases displaying sperm morphological defects were identified [15]. One of the mutations, R811H, is located in the enzymatic carboxypeptidase A domain. We also identified that *AGTPBP1* is specifically expressed during sperm head and tail formation, as seen in human testicular sections. In the same study, we also knocked out (KO) the entire carboxypeptidase A domain of AGTPBP1 in mice and observed severe sperm morphological defects, accompanied by abnormal tubulin polyglutamylation and reduced Δ2-tubulin levels [15].

Our previous mouse knockout study suggests the involvement of AGTPBP1 in sperm differentiation; however, it remains unclear if the R811H mutation observed in teratozoospermic patients would result in the same morphological defects in mice. In this study, we asked if a single nucleotide substitution, R791H, in mice (corresponding to the human R811H mutation) in the *Agtpbp1* gene would result in similar morphological defects in mice. For this, we generated *Agtpbp1*^*R791H/R791H*^ mice and analyzed sperm for morphological defects.

## Methods

### Generation of* Agtpbp1*^*R791H/R791H*^ knock-in mice

All animal procedures were approved by the Fu Jen Catholic University Institutional Animal Care and Use Committee (Approval No: A10979, approved on 03/18/2021). To create the *Agtpbp1* knock-in mouse model using the CRISPR/Cas9 system, we designed an sgRNA sequence (CAGACCATGGTGGATC CGTATGGGCACT) targeting exon 18 at c.2355. This site corresponds to the human p.R811H site in the carboxypeptidase domain of the AGTPBP1 human protein. The experiment was conducted in C57BL/6 mouse embryonic stem cells, following our previous studies [15, 27]. Targeted clones containing the desired allele were identified and validated by PCR using primers VF1 (5′-GGCTTCTGAGGGTTAATGGAG-3′) and VR1 (5′-TGGGGCTGAGTAGGGTCTAA-3′), followed by sequencing analyses. After injection into C57BL/6J blastocysts and implantation into pseudopregnant female mice, the resulting male chimeras were bred with wild-type females to produce *Agtpbp1*^+*/R791H*^ mice. Semen analysis parameters, including sperm concentration, motility, and morphology, were assessed and compared between wild-type male mice (*n* = 9) and *Agtpbp1*^*R791H/R791H*^ knock-in mice, both aged 3 months. For each analysis, a minimum of 200 sperm were evaluated. Statistical comparisons were performed using the *t*-test, with significance levels set at *p < 0.05 and ***p < 0.0001.

### Human sperm collection

The human study was conducted with approval from the Ethics Committees of Cathay General Hospital (IRB Approval Nos.: CGH-P102031 and CGHFJU-105006), and written informed consent was obtained from all participants. Semen samples were collected via masturbation following 3–5 days of sexual abstinence. After liquefaction, semen analysis was performed in accordance with the World Health Organization (WHO) 2010 guidelines [28].

### Murine sperm collection

Mature murine spermatozoa were harvested from the vas deferens and epididymis of 3-month-old C57BL/6 male mice. Following euthanasia, the vas deferens and epididymis were carefully excised from the mice. (1) Vas deferens collection: each vas deferens was placed in a sterile 35 mm Petri dish, and sperm were flushed out using a 21-gauge syringe containing 0.5 mL of pre-warmed human tubal fluid (HTF) medium. The dish was then incubated at 37 °C for 10 min to promote sperm motility. Subsequently, the sperm-containing medium was transferred to a 1.5-mL microcentrifuge tube and immediately used to prepare wet mounts for the initial assessment of sperm motility and morphology prior to fixation. (2) Epididymal sperm collection: each epididymis was placed in a separate 35 mm Petri dish containing 1 mL of pre-warmed HTF medium. The tissue was minced into ~ 1 mm^3^ fragments using fine scissors and incubated at 37 °C for 10 min to allow sperm to swim out into the medium. The supernatant was then collected and transferred to a fresh 1.5-mL microcentrifuge tube for sperm counting using a hemocytometer. (3) Storage: following the initial assessment, the remaining sperm samples were spread onto microscope slides and air-dried. Finally, the slides were stored at −80 °C until use in immunostaining assays.

### Fluorescence staining

The slides were permeabilized using 0.1% Triton X-100, rinsed twice with Tris-buffered saline (TBS), and subsequently incubated with 4′,6-diamidino-2-phenylindole (DAPI) for nuclear DNA staining and MitoTracker™ Red (Thermo Fisher Scientific, Cat. No. M7512) for mitochondrial staining, highlighting the midpiece region. Stained spermatozoa were visualized and imaged using an Olympus BX60 fluorescence microscope (Tokyo, Japan). All procedures were conducted as described in our earlier studies [29].

## Results

### Established* Agtpbp1*^*R791H/R791H *^knock-in (KI) mice

**Fig. 1:**
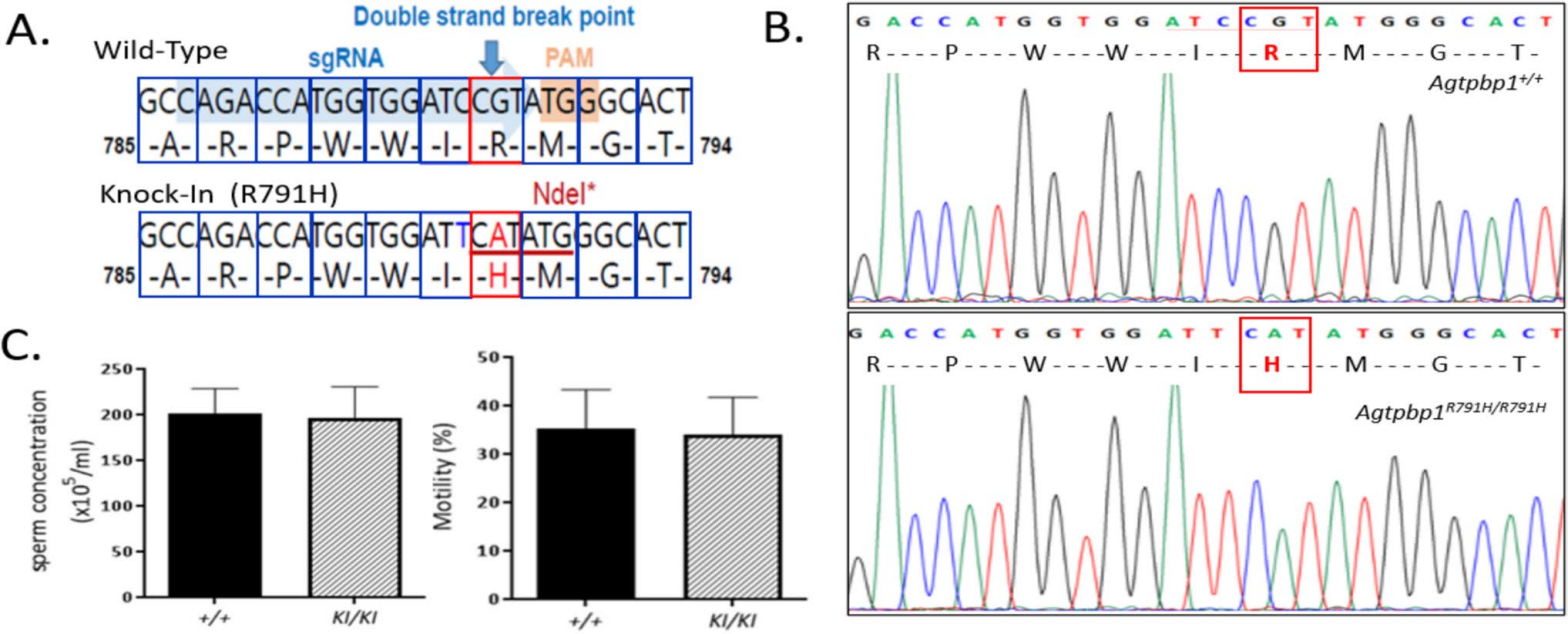
Generation of *Agtpbp1*^*R791H/R791H*^ knock-in mice and sperm analysis. **A** The sgRNA sequence (GACCATGGTGGATCCGT) targeting murine exon 18 of the *Agtpbp1* gene was designed to generate *Agtpbp1*^R791H/R791H^ knock-in mice using the CRISPR/Cas9 system. The blue arrow indicates the double-strand break site, and the single nucleotide change (CGT (R) → CAT (H)) is shown. NdeI: NdeI restriction enzyme site. **B** Sanger sequencing confirms the specific CGT → CAT (Arg791His; R791H) mutation in *Agtpbp1*^R791H/R791H^ knock-in mice, compared to the wild-type mice. **C** Comparison of different sperm parameters, including sperm concentration and sperm motility. Sperm were collected from the vas deferens of wild-type male mice and *Agtpbp1*^*R791H/R791H*^ knock-in mice

To confirm the effects of the specific mutation site (R811H) in the *AGTPBP1* gene on sperm morphology, we generated *Agtpbp1*^*R791H/R791H*^ knock-in (KI) mice using the CRISPR/Cas9 system. An sgRNA sequence (GACCATGGTGGATCCGT) targeting the c.2355 position (p.R791H) in murine exon 18 of the *Agtpbp1* gene, which corresponds to the human p.R811H mutation in the critical carboxypeptidase domain of the *Agtpbp1* protein, was designed. After transfecting the sgRNA and Cas9 into mouse embryonic stem cells, Cas9 recognized the PAM (Protospacer Adjacent Motif) and cleaved the specific site, three base pairs upstream of the PAM site (Fig. 1A). The mutated sequence (CGT > CAT; R > H) replaced the wild-type sequence through homology-directed DNA repair (Fig. 1A). After genotyping the clones, the R791H mutated clone was selected for microinjection into C57BL/6 J blastocysts to generate chimeric mice. After breeding, *Agtpbp1*^+*/R791H*^ knock-in (KI) mice were produced. Through cross-mating, wild-type and *Agtpbp1*^*R791H/R791H*^ knock-in (KI) mice were generated and confirmed by genotyping (Fig. 1B). Fertility testing was performed by mating *Agtpbp1*^*R791H/R791H*^ mutant males with wild-type females. The average litter size produced by mutant males showed no statistically significant difference compared to that of wild-type controls (*n* = 4, *p* > 0.05). A detailed sperm analysis was performed to compare *Agtpbp1*^*R791H/R791H*^ knock-in (KI) mice with wild-type mice. No significant differences were observed in sperm concentration or motility (Fig. 1C).

### R791H substitution affects sperm formation

**Fig. 2:**
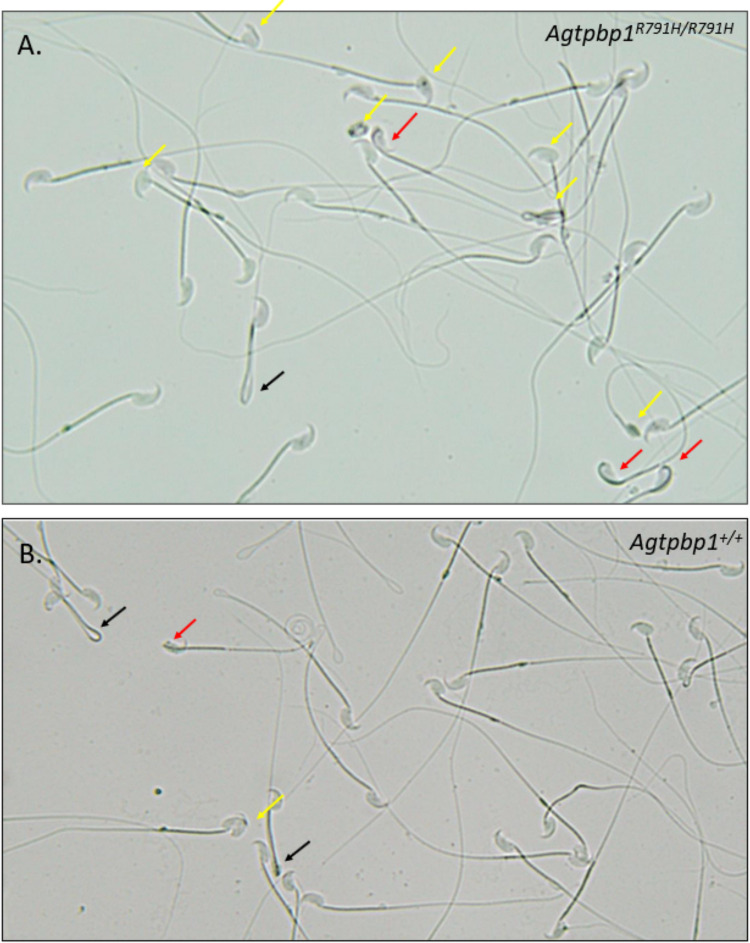
Sperm morphological defects in the *Agtpbp1 *^*R791H/R791H*^ knock-in mice. **A** Sperm from *Agtpbp1 *^*R791H/R791H*^ knock-in mice exhibit multiple morphological abnormalities, including incomplete head differentiation (yellow arrows), heads bent and attached to the midpiece (red arrows), and tails bent at the annulus, located at the end of the midpiece (black arrows). **B** Representative images of sperm from wild-type mice are shown for comparison

To assess the effect of the R791H substitution on sperm morphology in the *Agtpbp1*^*R791H/R791H*^ knock-in (KI) mice, mature sperm were isolated and evaluated for morphological defects. The sperm morphology analysis revealed a significant increase in the percentage of morphologically abnormal sperm in the *Agtpbp1*^*R791H/R791H*^ KI mice in comparison to the wild-type controls (Fig. 2; Table 1). One of the major defects was the presence of sperm heads with incomplete differentiation, some of which retained a portion of the midpiece over the head region (KI 20.220 ± 0.794% vs. WT 12.240 ± 1.733%; *p* < 0.05) (Fig. 2, yellow arrows). Another prominent defect was the abnormal attachment of the head to the midpiece at an inappropriate angle (Fig. 2, red arrows), which was observed at a significantly higher frequency in the R791H mutant mice in comparison to the wild-type controls (KI 10.080 ± 1.361% vs. WT 6.928 ± 0.480; *p* < 0.05). A third major defect involved sperm with tails completely bent at the midpiece, forming a hairpin-like structure (Fig. 2, black arrows). However, this particular abnormality was also observed in the wild-type mice (KI 9.007 ± 1.277% vs. WT 10.050 ± 1.703%; *p* > 0.05). Overall, the percentage of all sperm defects was significantly higher in the R791H mutant mice in comparison to the wild-type mice (KI 39.320 + 1.580% vs. WT 29.60 + 2.716; *p* < 0.05) (Table 1). These findings suggest that the R791H point mutation in the *Agtpbp1* gene impairs AGTPBP1 function, leading to structural abnormalities in sperm head and tail.

**Table 1:** Comparison of sperm defects between *Agtpbp1*^*R791H/R791H*^ and control mice

Defects	KI mice (%)	Control mice (%)	*p*-value
Incomplete head differentiation	20.220 ± 0.794	12.240 ± 1.733	0.0008***
Head bent and attached to the midpiece	10.080 ± 1.361	6.928 ± 0.480	0.0496*
Tail bent at the annulus, the end of the midpiece	9.007 ± 1.277	10.050 ± 1.703	0.6070
Total	39.320 ± 1.580	29.60 ± 2.716	0.0360*

^*^p < 0.05; ***p < 0.001

### Sperm exhibiting head defects from* Agtpbp1*^*R791H/R791H*^(KI) mice displayed abnormal aggregation of polyglutamylated tubulin in the head region

**Fig. 3:**
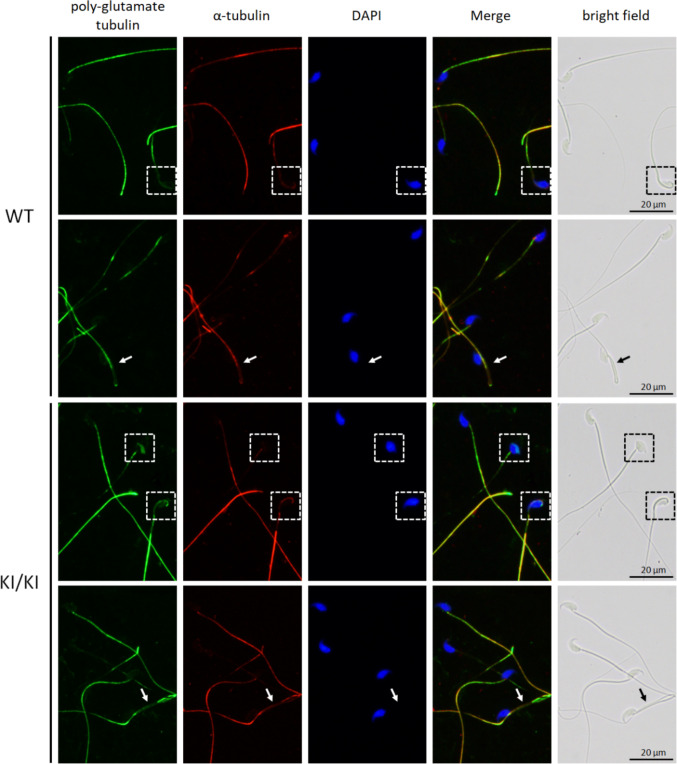
Polyglutamylated tubulin localization in *Agtpbp1* R791H (KI) sperm exhibiting morphological defects, including malformed heads and bent tails. Upper panel: wild-type sperm. Lower panel: knock-in (KI/KI) sperm. Lane 1 to Lane 5: immunofluorescence staining using anti-polyglutamylated tubulin antibody, anti-α-tubulin antibody, DAPI (DNA stain), merged image (Lane 1–Lane 3), and the corresponding bright-field image

To determine whether the *Agtpbp1* R791H mutation affected the molecular function of tubulin de-polyglutamylation in the knock-in (KI) sperm, immunofluorescence staining was performed using an anti-polyglutamylated tubulin antibody. In wild-type sperm, polyglutamylated tubulin signals were predominantly localized to the sperm tail (Fig. 3, upper panel). In morphologically abnormal wild-type sperm—such as those with misshapen heads (dashed squares) or bent tails (arrows)—a reduction in polyglutamylated tubulin staining was observed. In the *Agtpbp1* R791H KI sperm, abnormal accumulation of polyglutamylated tubulin signals was detected in the malformed sperm heads (Fig. 3, lower panel; dashed squares), whereas no significant changes in polyglutamylated tubulin signals were observed in the bent tail regions (arrows). These findings suggest that the R791H mutation in *Agtpbp1* partially disrupts its molecular function in regulating tubulin de-polyglutamylation during sperm development.

### R791H mutation does not affect AGTPBP1 localization or stability

**Fig. 4:**
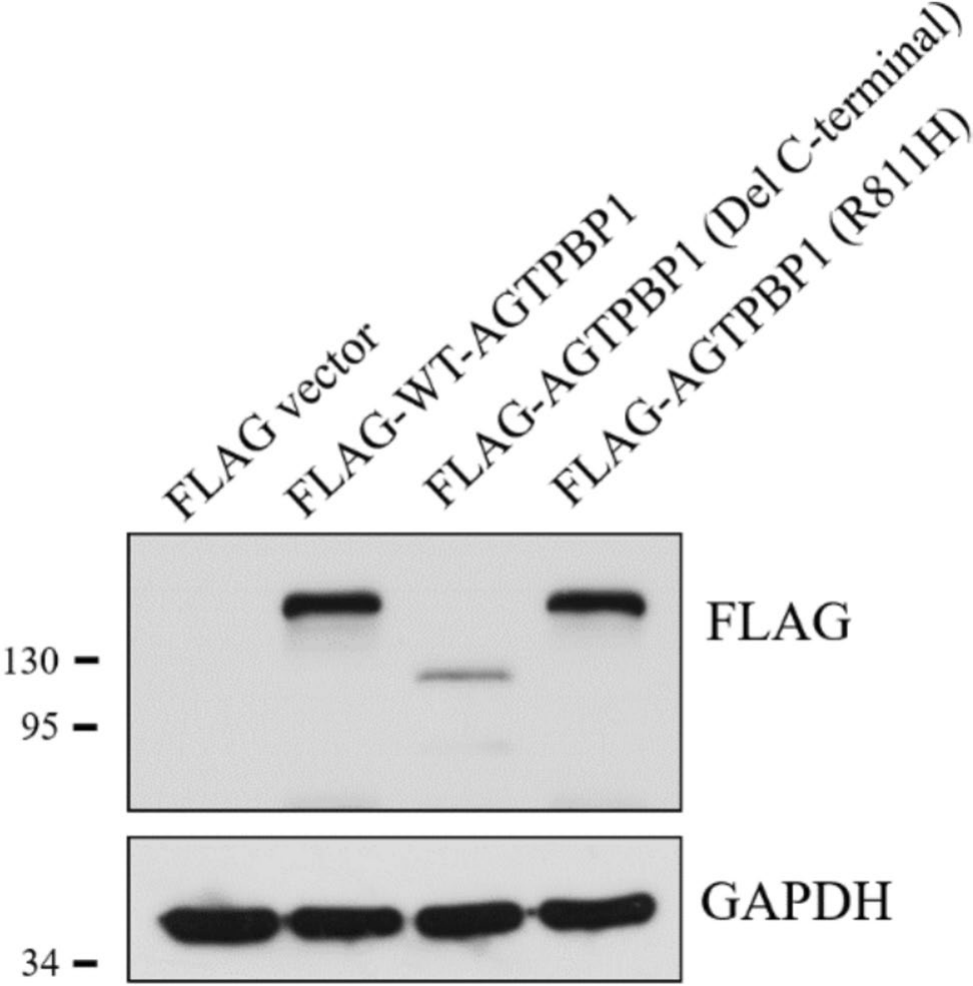
Transient expression of *Agtpbp1* R791H does not alter protein stability. A549 cells were transfected with expression vectors encoding wild-type *Agtpbp1*, a truncated form lacking the C-terminal carboxypeptidase A domain, or the R791H mutant. Western blot analysis was performed using an anti-FLAG antibody. Lanes 1–4: cells were transfected with (1) empty FLAG vector (FLAG-vector), (2) FLAG-tagged wild-type full-length AGTPBP1 (FLAG-WT-AGTPBP1), (3) FLAG-tagged AGTPBP1 with a C-terminal deletion (FLAG-AGTPBP1(Del C-terminal)), and (4) FLAG-tagged AGTPBP1 carrying the R791H mutation (FLAG-AGTPBP1(R791H))

To investigate whether the *Agtpbp1* R791H mutation affects AGTPBP1 protein stability or localization, we performed immunofluorescence staining using an anti-AGTPBP1 antibody on sperm from both wild-type and knock-in (KI) mice. The results showed the same localization as the wild type, indicating that the R791H mutation does not alter protein subcellular localization. To further assess the potential impact on protein stability, we constructed expression vectors for wild-type Agtpbp1, a truncated form of Agtpbp1 lacking the C-terminal carboxypeptidase A domain, and the *Agtpbp1* R791H mutant. Western blot analysis revealed that the expression levels of the wild-type and R791H mutant AGTPBP1 proteins were comparable, whereas deletion of the C-terminal domain led to a marked decrease in protein amount (Fig. 4). According to these results, we suggest that the R791H mutation does not impair AGTPBP1 protein stability. Taken together, our findings reveal that the R791H mutation does not affect the protein localization or stability of AGTPBP1.

### Comparative analysis of sperm morphological abnormalities in human* AGTPBP1*^*R811H*^and murine* Agtpbp1*^*del*^and* Agtpbp1*^*R791H*^models

**Fig. 5:**
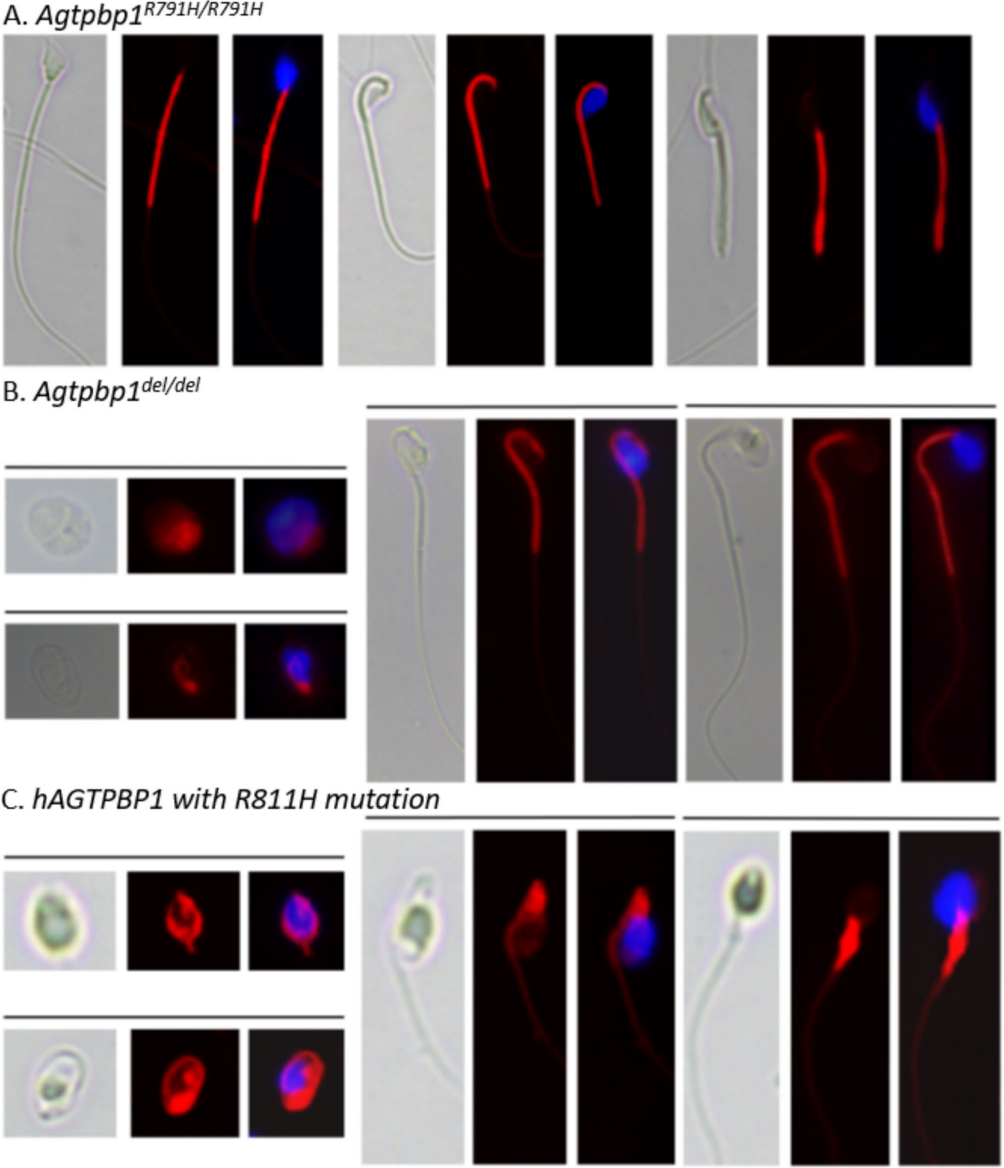
Comparative analysis of sperm morphological defects in human and mouse models with the AGTPBP1/Agtpbp1 mutation. Fluorescence staining of spermatozoa from *Agtpbp1*^*R791H/R791H*^ knock-in mice (**A**), *Agtpbp1*^*del/del*^ knockout mice (**B**), and a human subject carrying the R811H mutation in *AGTPBP1* (**C**), showing multiple morphological abnormalities. Mitochondria in the midpiece were stained with MitoTracker™ Red (red) and nuclei with DAPI (blue). **A** Sperm from *Agtpbp1*^*R791H/R791H*^ mice display incomplete head differentiation (left panel), heads bent and attached to the midpiece (middle panel), and tails bent at the annulus region (right panel). **B** and **C** Sperm from *Agtpbp1*^*del/del*^ knockout mice and the human R811H carrier exhibit similar abnormalities, including failure of tail elongation (left panel), heads bent and attached to the midpiece (middle panel), incomplete head differentiation (right panel). Images were captured at ×400 magnification

To investigate the impact of *AGTPBP1/Agtpbp1* mutations on sperm morphology, mature spermatozoa were collected from both *AGTPBP1*^*R811H*^ human subjects and genetically modified *Agtpbp1*^*del/del*^ and *Agtpbp1*^*R791H/R791H*^ mice, followed by fluorescence staining and microscopic evaluation. In *Agtpbp1*^*R791H/R791H*^ knock-in (KI) mice, a prominent defect was observed in sperm head morphology, characterized by abnormal shaping due to incomplete head differentiation (Fig. 5A, left; Table 1). Additionally, other morphological abnormalities included sperm with heads bent and abnormally attached to the midpiece (Fig. 5A, middle), as well as sperm with tails completely bent at the midpiece, forming a hairpin-like structure (Fig. 5A, right). In the *Agtpbp1*-deleted mice generated in our previous study [15], the defects were more severe: on average, 37% of sperm exhibited complete failure in tail formation, indicating a developmental defect during spermiogenesis (Fig. 5B, left). Even among elongated sperm, heads bent and attached to the midpiece (Fig. 5B, middle)**,** and the morphological structural defects in the head (Fig. 5B, right) were frequently observed. When comparing the human subject with the genetically modified mouse models, the individual carrying the heterozygous AGTPBP1 R811H mutation, with no other known pathogenic variants associated with spermatogenic failure, exhibited semen parameters characterized by a reduced sperm count (12 × 10^6^/mL), decreased motility (31%), and a high proportion of morphological abnormalities (97%). These findings were highly consistent with the phenotypes observed in Agtpbp1-deficient mice. These morphological phenotypes include the complete failure of tail formation (Fig. 5C, left), heads bent and attached to the midpiece (Fig. 5C, middle), and severely misshapen heads (Fig. 5C, left). This phenotypic concordance across species supports the potential pathogenic role of the R811H mutation in sperm-head and tail formation.

## Discussion

In our previous human study, we identified an R811H mutation in the carboxypeptidase A domain of the *AGTPBP1* gene in a teratozoospermic case [15]. Using a knockout mouse model, we further demonstrated that deletion of this functional domain led to a significant reduction in sperm count and motility, accompanied by severe morphological defects in both the sperm head and tail. At the molecular level, the loss of the carboxypeptidase A domain impaired enzymatic activity, resulting in abnormal tubulin polyglutamylation and a marked reduction in Δ2-tubulin levels, both critical for proper sperm head condensation and tail elongation [30]. In the present study, we asked if the R791H mutation in the *Agtpbp1* gene (homologous to the human R811H mutation) would also disrupt sperm head and tail formation in vivo*.* For this, we generated *Agtpbp1*^*R791H/R791H*^ mice using the CRISPR/Cas9 system. It was observed that sperm from the *Agtpbp1*^*R791H/R791H*^ mice exhibited similar defects as exhibited by those with the complete deletion of the *AGTPBP1* carboxypeptidase A domain and human males carrying the R811H mutation in the *AGTPBP1* gene, but at a much lesser frequency (Fig. 5 and Table 2).

**Table 2:** Comparison of the effects on sperm in response to *AGTPBP1/Agtpbp1* mutations in humans and mice

	Cases with mutated *AGTPBP1* R811H	*Agtpbp1*^*del/del*^ mice	*Agtpbp1*^*R791H/R791H*^ mice
AGTPBP1 amount	Decreased AGTPBP1	Loss of AGTPBP1	N/A
Sperm concentration	12 × 10^6^ (WHO ref limit:15 × 10^6^)	4.7 × 10^5^ (WT:184.3 × 10^5^)	Normal
Sperm motility	31% (WHO ref limit: 40%)	6% (WT: 46%)	Normal
Normal sperm morphology	3% (WHO lower limit: 4%)	3% (WT: 76%)	61% (WT:70%)
Deglutamylase enzymatic activity	N/A	Decreased	N/A
Delta (△)−2 tubulin amount	N/A	Decreased	N/A

*N/A* not available, *WT* wild-type

As shown in our previous study, AGTPBP1 shows significant expression in murine round spermatids, following which it slowly shifts to the post-acrosomal region and to the manchette structure, which facilitates sperm-head formation. In elongating spermatids, AGTPBP1 moves to the sperm midpiece during differentiation, leaving almost no AGTPBP1 protein in the sperm head [15]. Specifically, AGTPBP1 protein is localized at two points: immediately below the head and at the end of the midpiece. The two major sperm defects seen in the R791H mutant mice included incomplete head differentiation in some sperm and sperm heads fully bent and attached to the midpiece in other sperm, corresponding to the two points of AGTPBP1 localization in sperm. It is interesting to note that the incomplete sperm head differentiation phenotype seen in these mice is exactly the same as reported in our previous study, where part of the midpiece can be seen over the sperm head (Fig. 2A), suggesting incomplete differentiation of spermatids into spermatozoa. While this defect was occasionally seen in the wild-type mice, its frequency was significantly increased in mutant mice (Table 1). It is also worth noting that the same sperm-head differentiation defect was seen in human patients carrying the R811H mutation, as indicated by AGTPBP1 staining over the sperm head in these patients [15]. These defects are consistent with the role of this protein in tubulin processing and sperm head and tail formation.

Sperm from the *Agtpbp1* R791H knock-in (KI) mice exhibit a higher rate of morphological abnormalities. Several molecular mechanisms may contribute to this phenotype. First, we examined whether the R791H mutation affects AGTPBP1 localization. Immunofluorescence revealed similar localization patterns in the wild-type and KI sperm, indicating no effect on subcellular distribution. Second, overexpression of WT-AGTPBP1 and AGTPBP1-R791H showed comparable protein expression levels (Fig. 4), indicating that the mutation does not affect AGTPBP1 stability. Third, as reported in our previous study [15], the R791H substitution lies within the conserved carboxypeptidase A domain of AGTPBP1. Structural modeling predicts this substitution to disrupt local hydrogen bonding interactions with neighboring residues—such as glutamine (Gln), leucine (Leu), and isoleucine (Ile)—potentially impairing the structural integrity of the domain. Our findings indicate that the R791H mutation does not affect AGTPBP1 stability or localization, but impairs its enzymatic function in tubulin de-polyglutamylation. These findings support the notion that the R791H variant represents a hypomorphic allele—retaining partial function yet insufficient for normal sperm morphogenesis. Similar partial phenotypes have also been observed in missense mutation models of other sperm structural proteins. For instance, CFAP43 and CFAP44 missense variants lead to MMAF with less severe flagellar defects compared to their corresponding knockout models, suggesting that these mutations may retain partial protein function [31, 32]. This supports our interpretation that the *Agtpbp1* R791H variant may act as a hypomorphic allele.

Sperm from the *Agtpbp1*^*R791H/R791H*^ knock-in mice exhibited morphological changes at a lesser frequency in comparison to the mice lacking the entire carboxypeptidase A domain of AGTPBP1 or infertile men carrying the R811H mutation. Several factors could explain this discrepancy. First, although the *AGTPBP1* sequence between mouse and human is highly conserved [33], the *Agtpbp1*^*R791H/R791H*^ mutation may not precisely correspond to the human R811H mutation site [15]. Second, the *Agtpbp1*^*R791H*^ mutation in mice may not completely abolish carboxypeptidase function due to context differences in comparison to the R811H human mutation. In our previous study, Arg-to-His substitution at position 811 of AGTPBP1 in teratozoospermia cases resulted in AGTPBP1 protein instability and mislocalization [15]. We suggest that the *Agtpbp1*^R791H^ mutation may still preserve some protein function and stability. Third, significant differences in male reproductive biology, such as puberty onset and fertility duration, exist between humans and mice. Further, *Agtpbp1*^*R791H/R791H*^ knock-in mice were evaluated at 2–3 months of age, while reproductive senescence in mice corresponds to 21 months [34]. Fourth, the genetic background in mice could significantly affect the penetrance of genetic mutations. This discrepancy may contribute to the variations in phenotypic severity between mice and humans. Whether the *Agtpbp1*^*R791H/R791H*^ knock-in phenotype worsens with age remains to be investigated.

In conclusion, this study provides a more detailed in vivo characterization of the role of the R811H human mutation in the *AGTPBP1* gene. The R791H mutated mice showed exactly the same sperm morphological defects as seen in the *Agtpbp1* deleted mice, though in a lesser frequency. The localization of the defects in sperm head differentiation and tail bent at the midpiece is consistent with the role of AGTPBP1 in tubulin processing and sperm head and tail differentiation. The study contributes to our understanding of the molecular mechanisms underlying teratozoospermia and male infertility as a result of the R811H mutation in the *AGTPBP1* gene.

## Data Availability

Not applicable.
